# Tobacco Stained Fingers and Its Association with Death and Hospital Admission: A Retrospective Cohort Study

**DOI:** 10.1371/journal.pone.0138211

**Published:** 2015-09-16

**Authors:** Gregor John, Céline Louis, Amandine Berner, Daniel Genné

**Affiliations:** 1 Department of Internal Medicine, Hôpital neuchâtelois, 2300, La Chaux-de-Fonds, Switzerland; 2 Department of Internal medicine geriatrics and rehabilitation, Geneva University Hospitals (HUG), Gabrielle-Perret-Gentil 4, CH-1205, Geneva, Switzerland; 3 Department of Internal Medicine, Centre Hospitalier de Bienne, 2501, Bienne, Switzerland; West Virginia University, UNITED STATES

## Abstract

**Background:**

Among smokers, the presence of tobacco stains on fingers has recently been associated with a high prevalence of tobacco related conditions and alcohol abuse.

**Objective:**

we aimed to explore tobacco stains as a marker of death and hospital readmission.

**Method:**

Seventy-three smokers presenting tobacco-tar staining on their fingers and 70 control smokers were followed during a median of 5.5 years in a retrospective cohort study. We used the Kaplan-Meier survival analysis and the log-rank test to compare mortality and hospital readmission rates among smokers with and smokers without tobacco stains. Multivariable Cox models were used to adjust for confounding factors: age, gender, pack-year unit smoked, cancer, harmful alcohol use and diabetes. The number of hospital admissions was compared through a negative binomial regression and adjusted for the follow-up time, diabetes, and alcohol use.

**Results:**

Forty-three patients with tobacco-stained fingers died compared to 26 control smokers (HR 1.6; 95%CI: 1.0 to 2.7; p 0.048). The association was not statistically significant after adjustment. Patients with tobacco-stained fingers needed a readmission earlier than smokers without stains (HR 2.1; 95%CI: 1.4 to 3.1; p<0.001), and more often (incidence rate ratio (IRR) 1.6; 95%CI: 1.1 to 2.1). Associations between stains and the first hospital readmission (HR 1.6; 95%CI: 1.0 to 2.5), and number of readmissions (IRR 1.5; 95%CI: 1.1 to 2.1) persisted after adjustment for confounding factors.

**Conclusions:**

Compared to other smokers, those presenting tobacco-stained fingers have a high unadjusted mortality rate and need early and frequent hospital readmission even when controlling for confounders.

## Introduction

Tobacco smoking is the leading cause of preventable death around the world, killing nearly 6 million people every year [1]. Smokers have more than twice the mortality rate than non-smokers [2]. The 2014 US Surgeon General’s report established that tobacco-related mortality is caused by 21 diseases (including cancer, cardiovascular disease, diabetes, chronic obstructive pulmonary disease and pneumonia), but almost 20% of this excess mortality is believed to be associated with other diseases [3]. Moreover, tobacco-smoking has been proved to be an independent predictor of early hospital readmission in different settings, for instance among surgical patients [4–8], or patients suffering from psychiatric diseases (particularly schizophrenia) [9], or among patients with several other comorbid conditions (e.g. lung or cardiac conditions, or pancreatitis) [10–15]. Thus not only is tobacco smoking an economical burden, it is also associated with frequent hospital admissions and shorter life expectancy [1]. This burden increases with the number of cigarette smoked per day [16].

Tobacco stains on fingers are sometimes encountered among smokers, especially those smoking cigarettes without filters or starting their habit at a younger age [17]. This sign is associated with a high prevalence of tobacco-related conditions attributable to heavy smoking and alcohol abuse [17]. Therefore tobacco stains on fingers could indicate a high risk for subsequent hospital readmission and death. However little is known about such an association.

We aimed to determine the association between the presence of tobacco stains on fingers and the risk of hospital admission and death.

## Materials and Methods

### Study Design and Population

We performed a retrospective cohort study on a population of 143 smokers screened between March 2006 and January 2010 in a 180-bed community hospital in La Chaux-de-Fonds, Switzerland. Inclusion criteria, data collection and outcome definition have been previously reported [17]. Data on death and hospital admission were collected to the 30^th^ of June 2014.

We included consecutively all smokers presenting at least one tobacco stain on their fingers on admission to the departments of internal medicine, surgery, or the intensive care unit, irrespective of their initial diagnosis, and hospitalized in the period between March 2006 and December 2007 (6% of smokers). Controls were smokers admitted to the same department and at the same period of time through to January 2010, but having no tobacco stain on their fingers. They were initially meant to match patients with tobacco stains, as described in a previous report [17]. All participants provided written informed consent. Institutional Review Board of the hospital of La Chaux-de-Fonds approved the study.

### Outcomes and Measure

The primary outcome was all cause mortality. Information on death was obtained from the Swiss national death registry, the public state hospital and by contacting each participant’s general practitioner.

Secondary outcome was the time before the first readmission in the only public state hospital, and the number of admissions during the follow-up course. We reviewed inpatient medical records for number of hospital admissions, the date of and reason for the first readmission (after inclusion). The reasons for the first readmissions were classified into the following five categories depending on the principal diagnosis on the medical chart: "Alcohol or psychiatry", "respiratory", "cardiovascular", "oncology" and "miscellaneous". Alive participants who were not admitted to the hospital and had no contact with their general practitioner were considered as lost to follow-up, and were censored from the last visit date.

A single 30 minutes standard interview was performed for each participant to obtain medical history, and perform a physical examination and pulmonary function tests. A history of a tobacco-related disease and comorbid conditions, as well as tobacco smoking and alcohol habits were obtained from the patient’s interview and from the past and current medical records, classified according to the tenth revision of the International Statistical Classification of Diseases, (ICD-10) [18].

Vascular disease was defined by stroke, coronary heart disease, and peripheral arterial disease. The presence of coronary heart disease was defined by any of the following findings: a history of angina pectoris, myocardial infarction, coronary arterial bypass graft, arterial angioplasty or known vessel obstruction of 50% or more. Leg pain associated with an ankle brachial ratio index less than 0.9 or a procedure for arterial insufficiency defined a peripheral arterial disease. Chronic obstructive pulmonary disease was assessed from the patient’s interview, from the medical records and when forced expiratory volume in 1 second (FEV1) was 70% of the forced vital capacity on lung function test performed during the inclusion. We recorded all cancers (related or not to tobacco) except skin cancer.

The characteristics of smoking habits reviewed were: age at onset, number of packets smoked per day, unfiltered or low tar cigarettes and attempts at smoking cessation. The pack year unit (PY) was calculated as the number of packs smoked in a day multiplied by the number of years spent smoking. When the consumption was irregular from day to day we used the mean of the extremes as an estimate of the number of packs smoked daily. If the consumption was higher or lower for more than a year’s duration, the total cumulative PY was calculated as the sum of the different PYs to date.

Presence of a stain was assessed visually, without any specific instrument. We recorded the number and location of stains for each patient as well as the surface area in centimetres squared of the stain located on the finger skin only. The intensity of all stains was graded using a standardised visual scale from 1 to 5 depending on the colour (pale yellow to intense brown) [17].

We considered harmful alcohol use when high alcohol intake—more than 14 standard drinks/week for women and more than 21 standard drinks/week for men (140 and 210 g of alcohol, respectively)- was associated with documented withdrawal, medical consequences (neurological, cardiac, hepatic or pancreatic) or erythrocyte macrocytosis (mean corpuscular volume greater than 98 fL) not otherwise explained [17].

### Statistical Analysis

We calculated that 143 participants (51% presenting tobacco-stains on fingers) could detect a hazard ratio of 1.6 between groups with a statistical power of 80% and an alpha error of 5% (Freedman method).

We used Kaplan-Meier survival analysis and unweighted two-sided log-rank test to compare unadjusted mortality and time to the first hospital admission between smokers with and without tobacco stains. Time to the first hospital admission was censored in case of death before the closure date or occurrence of the outcome considered. Multivariable Cox models were used to adjust for the following potential confounding factors: age, gender, pack-year unit of cigarette smoked, cancer, harmful alcohol use, and diabetes. Choice of confounding factors was based on previous publications [17] and identified variables in univariate analyses. Proportional hazards assumption was verified using the Schoenfeld test.

All analyses were repeated among the restricted matched cohort (98 patients) previously published as a sensitivity analysis [17]. In this cohort, every patient with a tar stain on a finger was paired to one control at the time of inclusion, matched by age, gender, height, and pack-years smoked (49 pairs). Pairs were matched closely with up to 10% difference for each continuous variable. We then compared the predicted theoretical value of FEV1 (according to height, age and gender) of each pair to ensure a difference of <10% [17]. Stratified Cox model was used for time-event comparison.

We found an interaction between cancer and the association between tobacco stains and death or hospital admission. However this interaction was not statistically significant in adjusted models. We found no other interaction. Thus all models are given without interaction terms.

The multiple hospital readmissions were compared between patients with or without tobacco stains on fingers, through a negative binomial regression. This model was chosen because of an over dispersion of the data, not suitable for a Poisson regression and as indicated by the command *"countfit"* in STATA to compare count models [19]. This model was adjusted for follow-up time, harmful alcohol use, and diabetes.

Comparisons of characteristics among groups were performed using the chi-squared test or the Fisher exact test when appropriate for categorical variables. The two-sided t-test was used for continuous variables with normal distribution, and the Wilcoxon rank sum test for continuous variables not normally distributed. All analyses were performed using STATA statistical software, version 12.0 (StataCorp LP, Texas, USA).

## Results

### Participants

143 smokers were followed during a median period of 5.5 years (IQR: 1.8 to 7.4), which represents 335 patient-years with tobacco stains on fingers and 344 patient-years without stains. There were no participants lost to follow-up. Characteristics of participants are presented in Table 1. Patients with tobacco stains on fingers had higher PY smoked, harmful alcohol use, and proportion of cancer, but less diabetes.

**Table 1:** Baseline characteristics by presence of a tobacco stain on fingers.

Variables	All patients (143)	Tobacco-stain (73)	Controls (70)	p *
Median (IQR) age (years)	62 (50–71)	63 (52–71)	59.5 (50–71)	0.4 ^†^
Male N (%)	93 (65%)	53 (73%)	40 (57%)	0.05
Median (IQR) pack-years unit smoked ^‡^	50 (36–66)	54 (41–72)	45 (34–56)	<0.05 ^†^
Proportion of participant smoking unfiltered cigarettes	46 (32)	34 (47)	12 (17)	<0.001
Chronic obstructive pulmonary disease	90 (64)	52 (71)	38 (56)	0.06
Chronic kidney disease	16 (11)	8 (11)	8 (11)	0.9
Vascular disease ^§^	71 (50)	38 (52)	33 (47)	0.6
Oncologic disease	33 (23)	23 (31)	10 (14)	0.017
Hypertension	75 (52)	38 (52)	37 (53)	0.9
Diabetes	25 (17)	8 (11)	17 (24)	0.04
Psychiatric disease	52 (36)	31 (42)	21 (30)	0.1
Harmful alcohol use ^||^	57 (40)	39 (53)	18 (26)	<0.001

* Chi-squared test or the Fisher exact test

^†^ Wilcoxon rank sum test

^‡^ Number of packs smoked in a day multiplied by the number of years spent smoking or sum of the different PYs throughout life if the consumption was higher or lower for more than a year’s duration.

^§^ Vascular disease is composed from any of ischemic heart disease, stroke, or peripheral vascular disease.

^||^ Harmful alcohol use: high alcohol intake associated with documented withdrawal or medical consequences. IQR: interquartile range 25–75%

### Mortality Rate

**Fig 1:**
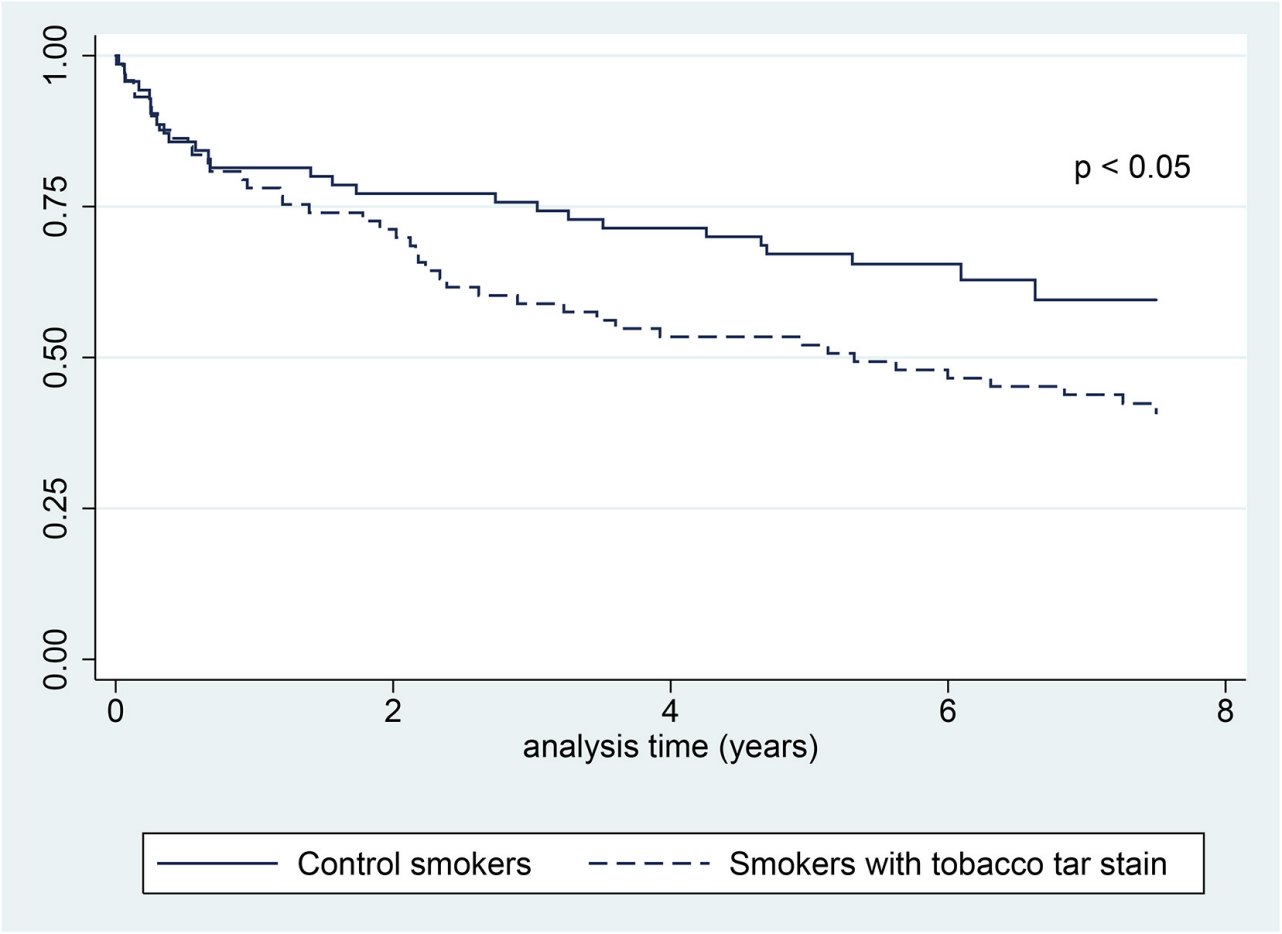
Kaplan-Meier curves illustrating overall survival for smokers with and without tobacco-tar stain on their fingers. P value determined with log rank test.

By the end of the follow-up period, 43 of the patients with stains on their finger and 26 controls were dead (p 0.009). Patients with tobacco stains had an unadjusted hazard ratio (HR) for death of 1.6 (95%CI: 1.0 to 2.7; p 0.048) (Fig 1). When adjusted for age, gender, pack-year unit smoked, and the comorbid condition (cancer, diabetes, and harmful alcohol use) the association between tobacco stains and death was not statistically significant (HR 0.8; 95%CI: 0.4 to 1.4; p 0.4). In the sensitivity analyses restricted to the matched cohort, the unadjusted HR for mortality (1.8; 95%CI: 0.8 to 3.9; p 0.1), and HR adjusted for diabetes, harmful alcohol use, and cancer (0.8; 95%CI: 0.2 to 3.0; p 0.7) were not statistically significant for tobacco stained smokers.

### Hospital Admission

**Fig 2:**
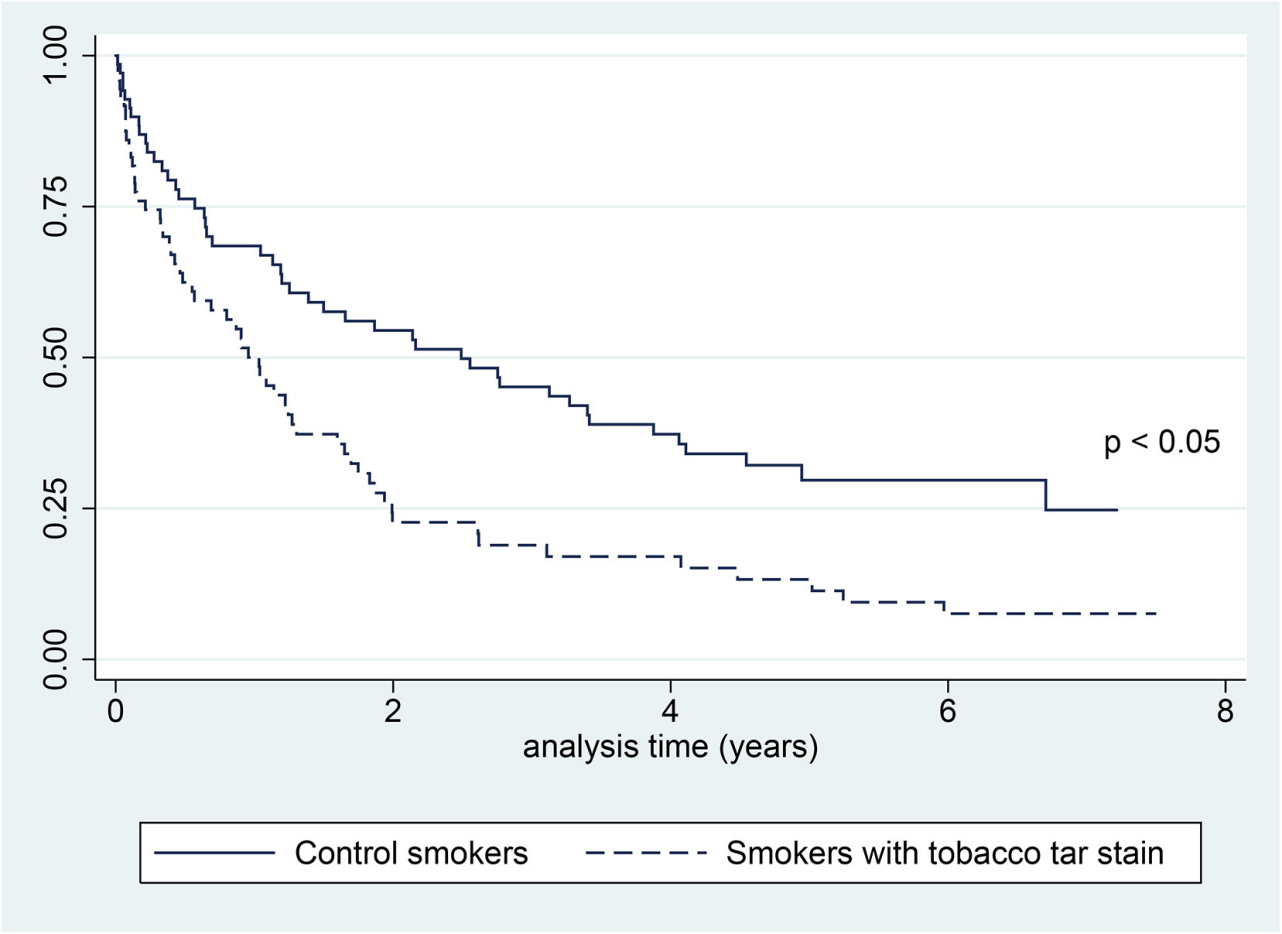
Kaplan-Meier curves for the first hospital readmission in smokers with stains on their fingers and control smokers. P value determined with log rank test.

Time to the first readmission was shorter for the patients with tobacco stains (HR 2.1; 95%CI: 1.4 to 3.1; p <0.001) (Fig 2). Half of these patients were readmitted after 1 year (95%CI: 0.5 to 1.3) versus 2.5 years (95%CI: 1.2 to 3.9) for the control group. In the multivariable model adjusted for age, gender, pack-year unit smoked, cancer, diabetes and harmful alcohol use, the association between tobacco stains and the first hospital admission was still statistically significant (adjusted HR 1.6; 95%CI: 1.0 to 2.5; p 0.03). When restricted to the matched case-control study, and adjusted for cancer, diabetes and harmful alcohol use, the HR was 2.4 (95%CI: 1.0 to 5.4; p 0.04).

Except for the category “alcohol and psychiatric”—which was more frequently encountered in patients with tobacco-stain—the frequency of reasons for readmission were similar for both groups (Table 2). The "alcohol or psychiatry" category encompasses admissions linked to alcohol related withdrawal, fall, pancreatitis, cirrhosis, and three suicide attempts.

**Table 2:** Death and hospital admission categories.

	All patients (143)	Tobacco-stain (73)	Controls (70)	p *
Number of death (%)	69 (48.2%)	43 (58.9%)	26 (37.1%)	<0.01
Yearly mortality rate	10.2% (8.0–12.9)	12.5% (9.3–16.8)	7.8% (5.3–11.4)	0.03^†^
Median (IQR) time to death, y	1.7 (0.3–3.5)	2.0 (0.5–3.6)^‡^	1.0 (0.3–3.5)^‡^	0.4^†^
Median (IQR) number of readmission	2 (0–4)	3 (1–4)	1 (0–3)	<0.01^†^
Median (IQR) time to the first readmission,y	0.9 (0.2–1.9)	0.8 (0.1–1.7)^‡^	1.2 (0.3–2.7) ^‡^	0.1^†^
Yearly hospital admission rate (95%IC)	36.7% (30.3–44.4)	55.0% (42.6–71.0)	25.7% (19.3–34.3)	<0.01^†^
**Readmission category**				
Alcohol or psychiatry	16 (11)	12 (16)	4 (4)	0.04
Respiratory	15 (10)	10 (14)	5 (7)	0.3
Cardiovascular	14 (10)	6 (8)	8 (11)	0.6
Oncology	11 (8)	6 (8)	5 (7)	1
Miscellaneous	51 (36)	25 (34)	26 (37)	0.7

* Chi-squared test or the Fisher exact test

^†^ Wilcoxon rank sum test

^‡^ Median time to death and median time to the first readmission in the restricted cohort were 1.9 years (IQR:0.7–4.9) and 0.9 year (IQR:0.3–1.7) for patients with tobacco-stained fingers and 0.7 year (IQR:0.3–3.0) and 1.1 years (IQR:0.2–2.8) for control smokers respectively.

95%CI: 95% confidence interval; IQR: interquartile range 25–75%; y: years.

During the follow-up period patients with tobacco stains had more hospital readmissions than those without stains 3 (IQR: 1 to 4) versus 1 (IQR: 0 to 3; p 0.008). In negative binomial regression adjusted for follow-up time, harmful alcohol use, and diabetes, participants with tobacco stains had 50% more hospital admissions (incidence-rate ratio 1.5; 95%IC: 1.1 to 2.1; p 0.02).

## Discussion

In our study, smokers with tobacco stains on their fingers had a high unadjusted mortality rate and experienced more frequent hospital readmissions. Half of these patients were readmitted at least once following the first year after the index hospital admission and half were dead after 5 years. Thus tobacco stains on fingers identify a vulnerable population of smokers that have poor outcomes. However, the association between tobacco stains and mortality is explained by the confounders–heavy smoking, high proportion of cancer and harmful alcohol use- and vanishes after adjusting for these factors.

Patients with tobacco stains compared to patients without stains are readmitted earlier and experience more frequent hospital readmissions. This difference persists after adjustment for potential confounders, like harmful alcohol use and cancer. Number of cigarette smoked [20–22] and alcohol abuse [23] seen among smokers with tobacco stains, have all been associated with frailty. Frailty is a known risk of death and hospital admission. Further studies should focus on measuring frailty for the subset of smokers with a tobacco stain.

The cause of readmission category "alcohol and psychiatry" was more frequent among smokers with tobacco stains. This reinforces a previous observation, that tobacco stains are linked to harmful alcohol use [17]. This association might have several possible explanations: deeper puff after alcohol consumption leaving more tar on finger's skin [24]; poor hand hygiene among patients with alcohol problems, or an addictive personality construct that impact both on smoking characteristics (deeper puff) and alcohol dependence [25–27].

Smoking and alcohol are known risk factors for hospital readmission in many settings [12, 28]. Besides, the number of rehospitalisation decreases when tobacco addiction is handled [29]. Intensive interventions during the hospital stay, that continue after discharge, with or without nicotine replacement therapy, is recognized as an effective strategy that can promote smoking cessation [30]. Such a strategy should be proposed to all hospitalized smokers. For the smokers with tobacco stains this could also represent an opportunity to enhance the transition from hospital to outpatient care and may avoid future readmission [18, 31, 32].

Several limitations of this study must be stressed out. First, all information was obtained at inclusion, and not reassessed thereafter. Stains could appear among the control smokers or vanish among the tobacco stained group. However stain is rather constant over time [17]. Third, stain was assessed visually and the interrater reliability is not known. Control smokers could have had unnoticed stains. Second, control smokers were selected to match participants with tobacco stains and thus could be unrepresentative of the general population of unselected smokers, especially in the outpatient setting. However these three points being pointed out, the selection of older, sicker, and heavy smoker controls, the possibility that stains appear between inclusion and adverse outcome, and the presence of subtle stain among control smokers could only blur the association, not create a spurious one.

## Conclusion

Tobacco stains pinpoint smokers with many tobacco related diseases and poor outcomes.

## Abbreviations

COPD: chronic obstructive pulmonary disease
HR: hazard ratio
IQR: Interquartile range
PY: The pack year unit smoked
